# The Prognostic Role of miR-375 in Head and Neck Squamous Cell Carcinoma: A Systematic Review, Meta-Analysis, and Trial Sequential Analysis

**DOI:** 10.3390/ijms26052183

**Published:** 2025-02-28

**Authors:** Mario Dioguardi, Gennaro Musella, Maria Eleonora Bizzoca, Diego Sovereto, Ciro Guerra, Pietro Laterza, Angelo Martella, Lorenzo Lo Muzio, Marina Di Domenico, Stefania Cantore, Andrea Ballini

**Affiliations:** 1 Department of Clinical and Experimental Medicine, University of Foggia, Via Rovelli 50, 71122 Foggia, Italy; mario.dioguardi@unifg.it (M.D.); gennaro.musella@unifg.it (G.M.); mariaeleonora.bizzoca@unifg.it (M.E.B.); diego_sovereto.546709@unifg.it (D.S.); ciro_guerra.556675@unifg.it (C.G.); pietro_laterza.572581@unifg.it (P.L.); lorenzo.lomuzio@unifg.it (L.L.M.); andrea.ballini@unifg.it (A.B.); 2 DataLab, Department of Engineering for Innovation, University of Salento, 73100 Lecce, Italy; angelo.martella@unisalento.it; 3 Department of Precision Medicine, University of Campania Luigi Vanvitelli, Via De Crecchio, 7, 80138 Naples, Italy; marina.didomenico@unicampania.it

**Keywords:** head and neck squamous cell carcinoma, laryngeal squamous cell carcinoma, oral squamous cell carcinoma, oropharyngeal squamous cell carcinoma, miR-375, oral cancer, non-coding RNA, microRNA

## Abstract

Head and Neck Squamous Cell Carcinoma (HNSCC) is a heterogeneous group of malignancies with poor survival outcomes, particularly in advanced stages. Identifying prognostic biomarkers could help improve patient management. miR-375, a small non-coding RNA, has been shown to influence tumor growth and immune responses, making it a candidate biomarker. This study aims to evaluate the role of miR-375 expression in predicting survival outcomes in HNSCC patients. A systematic review and meta-analysis were conducted according to PRISMA guidelines, incorporating data from six studies and the TGCA cohort, encompassing 452 patients. Fixed-effects models were applied to calculate aggregated hazard ratios (HRs) for overall survival (OS). Kaplan–Meier curves were analyzed using the Tierney method, and Trial Sequential Analysis (TSA) was performed to assess statistical power. Low miR-375 expression was associated with poorer OS, with an aggregated HR of 1.23 (95% CI: 1.10–1.37). Subgroup analysis showed consistent trends across oral and laryngeal squamous cell carcinoma. Sensitivity analysis confirmed these findings. TSA revealed that although the number of patients was sufficient, statistical power was insufficient to confirm a predefined risk reduction ratio (RRR) of 49%. Data from the TGCA cohort supported the meta-analysis findings, with an HR for OS of 1.32 (95% CI: 0.96–1.8). Low miR-375 expression is associated with worse survival outcomes in HNSCC patients, indicating its potential as a prognostic biomarker and therapeutic target. However, the retrospective nature of the included studies underscores the need for prospective research to validate these findings.

## Simple Summary

Head and Neck Squamous Cell Carcinoma (HNSCC) is a complex group of cancers with poor survival outcomes in advanced stages. Identifying reliable biomarkers could improve patient prognosis and guide treatment strategies. miR-375, a small non-coding RNA, has been implicated in regulating tumor growth, invasion, and immune responses across various cancers. This study explores the role of miR-375 as a prognostic biomarker in HNSCC by conducting a systematic review and meta-analysis, supplemented by a Trial Sequential Analysis. By examining data from six studies and the TGCA database, we aim to evaluate the association between miR-375 expression levels and survival outcomes. Our findings suggest that low miR-375 expression correlates with worse survival, indicating its potential as a prognostic marker and therapeutic target. These results provide a foundation for further research into miR-375’s role in cancer biology and its clinical application in improving HNSCC patient outcomes.

## 1. Introduction

Head and Neck Squamous Cell Carcinoma (HNSCC) encompasses a heterogeneous group of malignancies arising from the epithelial lining of mucosal surfaces. The primary anatomical sites affected include the oral cavity (oral squamous cell carcinoma, OSCC), the larynx (laryngeal squamous cell carcinoma, LSCC), the hypopharynx (hypopharyngeal squamous cell carcinoma, HSCC), the nasopharynx (nasopharyngeal carcinoma, NPC), and the oropharynx (oropharyngeal squamous cell carcinoma, OPSCC) [1,2,3].

HNSCC is the sixth most common cancer worldwide, with 960,000 new cases annually (4.8% of global cancer diagnoses), a five-year average survival rate of 30% for stages III and IV, and 450,000 deaths per year (4.9% of global cancer-related mortality) [4].

The primary risk factors for head and neck cancers are tobacco use and alcohol consumption, while HPV positivity, particularly for genotypes 16 and 18, is associated with OPSCC [5]. Generally, HPV positivity correlates with a better prognosis due to enhanced responsiveness to radiotherapy [6]. Growing attention has been given to the role of HPV in oropharyngeal cancer, highlighting the importance of awareness and prevention strategies in health education [7,8].

Tumorigenesis is a complex and multifactorial process involving genetic and epigenetic alterations as well as environmental influences [9,10], leading to disruptions in cellular proliferation, invasion, migration, apoptosis, and death. Some of these processes are partially mediated by alterations in microRNA (miR) expression [11,12].

miRs are small non-coding RNA sequences found in the transcriptomes of living organisms and certain DNA viruses. They consist of nucleotide sequences ranging from 18 to 22 bases. miRs regulate gene expression at transcriptional and post-transcriptional levels, modulating both physiological and pathological cellular activities [13].

In tumor tissues, miRs can exhibit altered expression patterns, being either upregulated (e.g., miR-21, miR-31) [14,15] or downregulated (e.g., let-7, miR-26a, miR-99a-5p) [16,17] compared to non-tumor tissues, highlighting their potential as prognostic biomarkers for survival. In the context of HNSCC, numerous studies have investigated the predictive potential of miR expression levels, focusing on either low or high expression [18], with 15 systematic reviews with meta-analyses specifically examining 64 miRs (e.g., miR-205, miR-429, miR-21, miR-375) [18,19]. Recent studies have highlighted the critical role of miR-375, demonstrating its significant influence on epithelial-mesenchymal transition (EMT) in human tumor epithelial cells. By driving this process, which promotes migration and metastasis, miR-375 emerges as a key factor in worsening patient prognosis [20].

MiR-375 is located on chromosome 2, specifically at chr2:219001645–219001708, and has a stem-loop structure: ccccGCGACGAGCCCCUCGCACAAACCggaccugagcguUUUGUUCGUUCGGCUCGCGUGAggc. It generates two mature forms: hsa-miR-375-3p (UUUGUUCGUUCGGCUCGCGUGAe) and hsa-miR-375-5p (GCGACGAGCCCCUCGCACAAACC) (https://www.mirbase.org/hairpin/MI0000783, access date: 7 December 2024) [21].

A recent study on miR-21, miR-155, and miR-375 identified that miR-375 exhibits anti-tumorigenic properties in OSCC [22,23], emphasizing its potential role in suppressing cancer progression. Moreover, its abundance in the serum of prostate cancer patients [24,25] and elevated levels in non-small cell lung cancer tissues [26] suggest its involvement in specific cancer pathways. In contrast, miR-375 is significantly downregulated in hepatocellular carcinoma [27], where it targets AEG-1 and suppresses liver cancer cell growth both in vitro and in vivo [28]. Similarly, its downregulation in gastric carcinoma [29], reinforces its tumor-suppressive role, as it appears to regulate gastric cancer cell proliferation via the JAK2 oncogene pathway.

Specifically, for HNSCC, a recent study by Dai et al. (2022) [30] revealed that downregulated miR-375 expression was observed in LSCC cells, whereas the overexpression of miR-375 inhibited cell viability and migration while promoting apoptosis in these cells.

Thus, reduced miR-375 expression in tissues or circulation may indicate the presence of malignancy and an unfavorable prognosis for many malignant tumors [31].

A recent systematic review and meta-analysis by Fang et al. (2022) [32]. on esophageal carcinoma (not classified as HNSCC) identified a hazard ratio (HR) for overall survival (OS) between high and low miR-375 expression of 0.50; 95% CI: 0.37–0.69, suggesting that upregulation may be associated with improved survival outcome.

Although these studies suggest a correlation between low miR-375 expression and reduced survival rates in various carcinomas, its specific role and true prognostic value in HNSCC need to be further clarified [33,34,35,36,37,38,39,40]. Therefore, our study aims to assess miR-375 as a prognostic biomarker for survival in HNSCC, exploring its potential as an individual marker or as part of a broader signature comprising non-coding RNAs to better elucidate its clinical significance and potential applications.

## 2. Methods

### 2.1. Protocol

The planning of this systematic review and meta-analysis was conducted following the recommendations of the Cochrane Handbook for Systematic Reviews of Interventions [10]. Our manuscript was prepared in accordance with the PRISMA (Preferred Reporting Items for Systematic Reviews and Meta-Analysis) guidelines [12]. The protocol was registered on the PROSPERO platform (International Prospective Register of Systematic Reviews) under the registration number PROSPERO 2025 CRD42025632228, available from https://www.crd.york.ac.uk/prospero/display_record.php?ID=CRD42025632228 (access date: 18 January 2025), before proceeding with article selection.

### 2.2. Eligibility Criteria, Information Sources, Risk of Bias, Search and Selection

The search for bibliographic sources from articles and reports focused on all randomized and non-randomized clinical trials, as well as prospective and retrospective studies, investigating the role of miR-375 in HNSCC and its clear correlation with prognostic survival indices and miR-375 expression.

The PICO question was as follows: Is there a correlation between prognostic survival indices—OS (overall survival), PFS (progression-free survival), RFS (recurrence-free survival), DFS (disease-free survival), CSS (cancer-specific survival)—and altered miR-375 expression in patients with HNSCC?

The specific points addressed were as follows:

(P) Participants: Patients with HNSCC;

(I) Intervention: Altered miR-375 expression in HNSCC;

(C) Control: HNSCC patients with low miR-375 expression;

(O) Outcome: Difference in survival prognosis between patients with low and high miR-375 expression in HNSCC.

The exclusion criteria for the systematic review were as follows:

✓ Studies that did not report data on tissue expression of miR-375 in HNSCC or its histological subtypes, such as LSCC, OPSCC, HSCC, and OSCC;
✓ Studies published in languages other than English;
✓ Studies that did not report prognostic survival indices;
✓ Literature reviews (considered only as bibliographic sources), case reports, and case series.

Articles on miR-375 presenting results for relative risk (RR), hazard ratio (HR), Cox regression, or Kaplan–Meier survival curves for HNSCC prognostic indices were considered potentially eligible for the meta-analysis.

The review process involved two reviewers (M.D. and G.M.). The search and selection process was divided into five steps:

Defining and deciding on the inclusion and exclusion criteria, databases, keywords, and the time frame for the search;

Independently conducting the search and selection of studies;

Removing duplicates using reference management software such as EndNote 8.0;

Selecting studies for inclusion;

Comparing the included studies and resolving any conflicts between the two reviewers with the assistance, if necessary, of a third and fourth reviewer (S.C. and A.B.).

The keywords used included the following: miR-375, miR-375 AND HNSCC, LSCC AND miR-375, OSCC AND miR-375.

The search was conducted across three databases—ScienceDirect, SCOPUS, and PubMed—and one registry: the Cochrane Central Trial. Additionally, Google Scholar (keywords: miR-375), gray literature sources such as Open Gray (keywords: miR-375), and references from previous systematic reviews on miRs and HNSCC were consulted.

Below is a complete list of the keywords used for the PubMed search:

✓ Search: (miR-375 OR microRNA-375) AND (HNSCC OR LSCC OR HSCC OR OSCC) Sort by: Most Recent (“miR-375”[All Fields] OR “microRNA-375”[All Fields]) AND (“hnsccs”[All Fields] OR “squamous cell carcinoma of head and neck”[MeSH Terms] OR (“squamous”[All Fields] AND “cell”[All Fields] AND “carcinoma”[All Fields] AND “head”[All Fields] AND “neck”[All Fields]) OR “squamous cell carcinoma of head and neck”[All Fields] OR “hnscc”[All Fields] OR “LSCC”[All Fields] OR “HSCC”[All Fields] OR “OSCC”[All Fields]. Translations: HNSCC: “hnsccs”[All Fields] OR “squamous cell carcinoma of head and neck”[MeSH Terms] OR (“squamous”[All Fields] AND “cell”[All Fields] AND “carcinoma”[All Fields] AND “head”[All Fields] AND “neck”[All Fields]) OR “squamous cell carcinoma of head and neck”[All Fields] OR “hnscc”[All Fields].

The literature search was completed on 2 December 2024, with a final update conducted on 10 December 2024.

The data to be extracted from the included articles were predetermined by the two reviewers and included the study’s first author, publication date, country where the research was conducted, type of squamous cell carcinoma, number of patients, clinical characteristics of the patients and tumors (age, gender, smoking habits, HPV-positive status, follow-up period, tumor grading and staging), the miRs studied, the cut-off value or type distinguishing low and high miR-375 expression, and the RR and HR values for various prognostic survival indices.

In cases where only Kaplan–Meier survival curves were available, the hazard ratio was calculated using the Tierney method [39], extracting data from the curves using the Engauge Digitizer 4.1 software, ver. 4.1 (an open-source, non-commercial project available at https://engauge-digitizer.software.informer.com/4.1/ accessed on 10 November 2024) and recorded in a dedicated Excel spreadsheet, which is available online as supplementary material to the publication by Tierney et al. [39].

Additionally, the TCGA (The Cancer Genome Atlas) database, containing a cohort of patients with HNSCC, was consulted to extract HR values related to prognostic indices associated with miR-375 expression. These values were excluded from the meta-analysis to avoid bias due to data heterogeneity, as the TCGA cohort included approximately 522 patients, which was four times larger than the largest population study.

The risk of bias in individual studies was assessed by two authors (M.D. and G.M.) using evaluation criteria derived from the REMARK (Reporting Recommendations for Tumor Marker Prognostic Studies) protocol. Studies with a high risk of bias were excluded from the meta-analysis [41].

Heterogeneity among studies was assessed using Higgins’ index (I^2^) and the Chi^2^ test. For the meta-analysis, particularly for calculating the aggregated HR or RR, the Reviewer Manager 5.4 software (Cochrane Collaboration, Copenhagen, Denmark) was used.

The quality of evidence was assessed using the online software GRADEpro Guideline Development Tool (GRADEpro GDT, Evidence Prime, Hamilton, ON, Canada, https://www.gradepro.org/, access date: 18 December 2024) [34].

The Trial Sequential Analysis (TSA) was performed using Stata Stata 13 ver. 13.1 software (StataCorp, College Station, TX, USA) with the implementation of R 4.2 software and the installation of the id-bounds and metacumbounds commands.

## 3. Results

### 3.1. Selection of Studies

The searches conducted in ScienceDirect, SCOPUS, PubMed, and Cochrane Central Trial yielded 3978 bibliographic sources. After removing duplicates, 2791 potentially eligible articles remained. Of these, only 59 articles underwent full-text screening, and only six fully met the inclusion and exclusion criteria. Consequently, the relevant extracted data from these six studies were included in the meta-analysis.

Additionally, gray literature searches (http://www.opengrey.eu, accessed on 1 November 2024), DANS EASY Archive, Google Scholar, and previous systematic reviews using “miR-375” as a keyword did not identify any further studies eligible for inclusion in the meta-analysis.

Furthermore, a TGCA analysis was performed using the Kaplan–Meier plotter database portal (https://kmplot.com/analysis/, accessed on 10 November 2024), and HR data were extracted.

**Figure 1:**
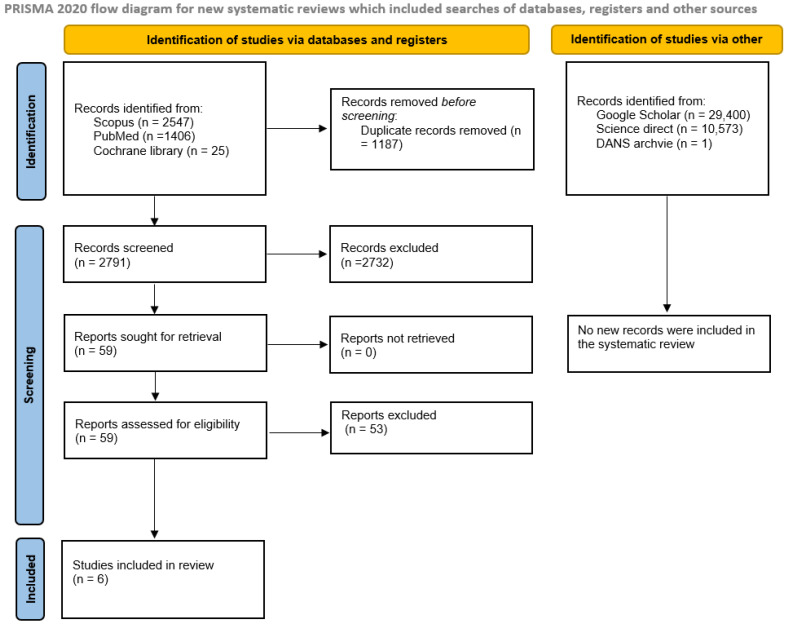
Entire selection and screening procedures are described in the PRISMA flowchart.

The complete procedure for the identification, selection, and inclusion of studies is outlined in the flowchart shown in Figure 1.

### 3.2. Characteristics of the Data and Included Studies

The systematic review included six articles, five of which were retrospective studies and one prospective study (Harris et al., 2012 [33]). The studies are as follows: Zhang et al., 2017 [34], Jia et al., 2015 [35] Hu et al., 2014 [36], Hudcova et al., 2016 [37], Yoon et al., 2014 [38].

The total number of patients with HNSCC included across the studies was 460, of which 452 were included in the meta-analysis. Among these, 334 cases were OSCC (326 of which were included in the meta-analysis), with 105 identified as TSCC (Tongue squamous cell carcinoma). LSCC was present in 89 patients, while OPSCC was reported in 37.

The most commonly used prognostic index was OS (overall survival), which was reported in all studies. DFS (disease-free survival) and RFS (recurrence-free survival) were provided as HR values only in the study by Hudcova et al., 2016 [37]. However, HR values for CSS (cancer-specific survival) and PFS (progression-free survival) were not reported in any of the six studies. Harris et al., 2012 [33] presented HR values for OS separately for three groups of patients (43 OSCC, 37 OPSCC, and 43 LSCC). These groups were analyzed independently in the meta-analysis as distinct cohorts (Table 1).

Survival data presented as Kaplan–Meier curves were extracted using the Tierney method [39], and the HRs obtained are reported in Table 2.

Regarding risk factors, two studies investigated HPV positivity (Yoon et al., 2014 [38] and Harris et al., 2012 [33]), identifying 34 HPV-positive and 91 HPV-negative patients out of 223, with HPV status unknown for 98 patients. Three studies investigated smoking status and alcohol consumption (Hu et al., 2014 [36], Harris et al., 2012 [33], and Yoon et al., 2014 [38]), reporting that 175 out of 261 patients were current or former smokers, and 72 out of 261 reported alcohol use. However, no data on the number of cigarettes smoked or the quantity of alcohol consumed were available in this systematic review.

Among the 400 total patients, 137 were female (Zhang et al. [34] did not report data on gender; consequently, its REMARK risk of bias assessment reflected a lower score compared to the other studies).

The estimated mean age of the patients was approximately 63.5 years. All six studies included were published between 2012 and 2017, with a mean follow-up range of 48 to 60 months. All prognostic survival indices included in the meta-analysis were extracted and are summarized in Table 2.

### 3.3. Risk of Bias in Studies

The risk of bias in the included studies was assessed using a classification derived from the REMARK guidelines [41]. Additionally, each parameter was evaluated as adequate, inadequate, or not assessable based on the REMARK guidelines. A score ranging from 0 to 3 was assigned to each factor according to the REMARK criteria (Table 3).

### 3.4. TGCA Cohort Analysis Results

**Figure 2:**
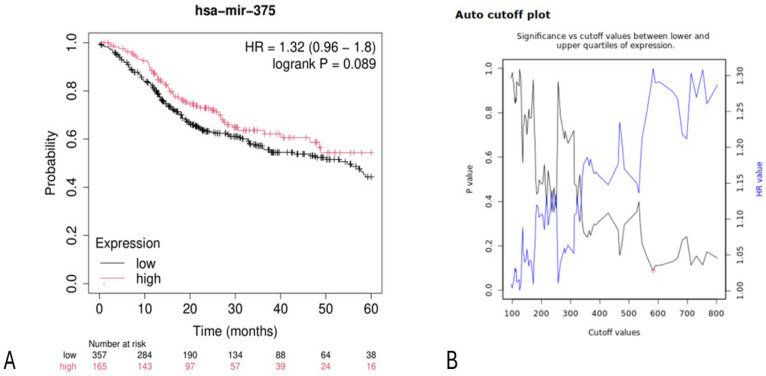
(**A**) Kaplan–Meier curves based on miR-375 expression levels for overall survival (OS) in HNSCC patients (TCGA cohort). Kaplan–Meier curves were generated using the public database and the Kaplan–Meier plotter web application (http://kmplot.com/analysis/, accessed on 16 December 2024). The curve and the associated data can be easily generated and reproduced through the portal. (**B**) Auto cut-off plot. Automatically generated cut-off plot using the Kaplan–Meier plotter web application, http://kmplot.com/analysis/, accessed on 16 December 2024. Significance vs. cut-off values between the lower and upper quartiles of expression are presented, with the red circle indicating the best cut-off.

The TGCA cohort analysis conducted via the Kaplan–Meier plotter database portal (https://kmplot.com/analysis/, accessed on 16 December 2024) on a cohort of 522 HNSCC patients generated Kaplan–Meier survival curves (Figure 2) comparing low and high miR-375 expression levels.

The portal is used to generate and visualize Kaplan–Meier plots, determine a cut-off value, and assign samples to one of two cohorts based on the best available cut-off.

To determine the best cut-off, the process is repeated using input variable values ranging from the lowest to the highest quartile, and Cox regression is calculated for each setting [42]. The most significant cut-off value was used as the optimal threshold to separate input data into two groups. Subsequently, the system provides a simple visual representation of this analysis, displaying the obtained *p*-values in relation to the selected cut-off values.

In cases where the generated cut-off values were ambiguous (e.g., multiple cut-off values yielded very low *p*-values), the value corresponding to the highest hazard ratio was selected. The calculation of multiple cut-off values resulted in the generation of multiple hypotheses. Therefore, in this configuration, the false discovery rate (FDR) was automatically calculated using the Benjamini–Hochberg method to adjust for multiple hypothesis testing [43].

The cut-off value distinguishing low and high miR-375 expression levels was automatically generated through the portal (Figure 2).

The bioinformatics analysis of miR-375 reported an HR for OS between low and high expression groups of 1.32 (0.96–1.8), with a log-rank *p*-value of 0.089. The median survival for the low-expression cohort was 14.33 months, while it was 19.33 months for the high-expression cohort.

A limitation of this analysis is that the log-rank test *p*-value is slightly above 0.05, indicating low statistical significance (Figure 2).

### 3.5. Meta-Analysis, Sensitivity Analysis, Subgroup Analysis, Publication Bias

The meta-analysis of the data was conducted using Review Manager 5.4 software (Cochrane Collaboration, Copenhagen, Denmark), which was also used to generate the forest plot and funnel plot images.

**Figure 3:**
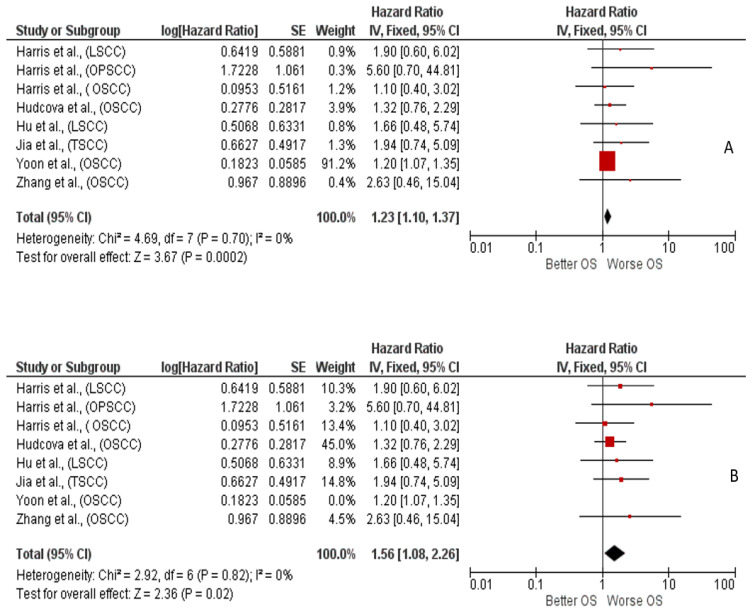
**(A**) Forest plot of the fixed-effects model of the meta-analysis: OS, HR = 1.23, 95% CI: [1.1, 1.37]; df = degrees of freedom; I^2^ = Higgins’ heterogeneity index (I^2^ < 50% indicates irrelevant heterogeneity; I^2^ > 75% indicates significant heterogeneity); C.I. = confidence intervals; P = *p*-value; SE = standard error. The studies included in the meta-analysis were: Harris et al., 2012 [33], Zhang et al., 2017 [34], Jia et al., 2015 [35], Hu et al., 2014 [36] Hudcova et al., 2016 [37], and Yoon et al., 2014 [38]. The graph for each study shows the lead author and year of publication, the hazard ratio (HR) with confidence intervals, the log HR standard error, and the weight of each study expressed as a percentage. The final value is expressed in bold with the corresponding confidence intervals. The black line indicates the position of the average value, and the light black diamond represents the measure of the average effect. (**B**) Sensitivity analysis: After excluding the study by Yoon et al., 2014 [38], which carried excessive weight in the analysis, the final effects consistently favor a worsening of OS in patients with low tissue expression of miR-375.

The meta-analysis focused on the HR for OS between low and high tissue expression of miR-375. Fixed effects were applied, and the log hazard ratio and standard deviation (SD) between the two groups (low and high expression) were calculated. The aggregated HR value was 1.23 [0.54, 1.54], indicating a slight worsening of OS in patients with low miR-375 expression. The black diamond in the forest plot, representing the effect size, narrowly missed the line of no effect (Figure 3A). The studies included in the meta-analysis were Harris et al., 2012 [33], Zhang et al., 2017 [34], Jia et al., 2015 [35], Hu et al., 2014 [36] Hudcova et al., 2016 [37], and Yoon et al., 2014 [38]. For Harris et al. [33], multiple patient cohorts were included, as these were stratified by HNSCC subtype. All studies crossed the line of no effect with their confidence intervals (Figure 3B).

Additionally, a sensitivity analysis was conducted by excluding the study by Yoon et al., which had an excessive weight in the meta-analysis due to narrower confidence intervals compared to the other studies. When this study was excluded, the HR for OS increased to 1.56 [1.08, 2.26], still indicating a worsening of OS in patients with low miR-375 expression.

A subgroup analysis was also conducted based on the type of HNSCC:

✓ Subgroup OSCC (including data related to TSCC).
✓ Subgroup LSCC (based on only two studies).
✓ Subgroup OPSCC (based on a single study).

**Figure 4:**
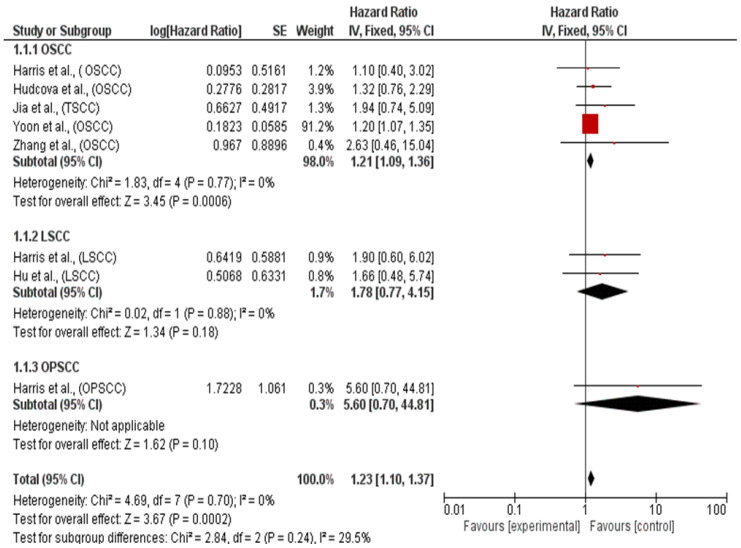
Forest plot of the fixed-effects model of the subgroup meta-analysis for OS, test for subgroup differences. Chi2 = 2.84. df (*p*-value = 0.24), I^2^ = 29.5%. The studies included in the meta-analysis were: Harris et al., 2012 [33], Zhang et al., 2017 [34], Jia et al., 2015 [35], Hu et al., 2014 [36] Hudcova et al., 2016 [37], and Yoon et al., 2014 [38].

The aggregated HR value for the first subgroup, OSCC, was 1.21 [1.09, 1.36], for the second subgroup, LSCC, it was 1.78 [0.77, 4.15], and for the third subgroup, OPSCC, it was 5.60 [0.70, 44.81]. The values depicted in Figure 4 did not deviate significantly from those shown in the main meta-analysis.

**Figure 5:**
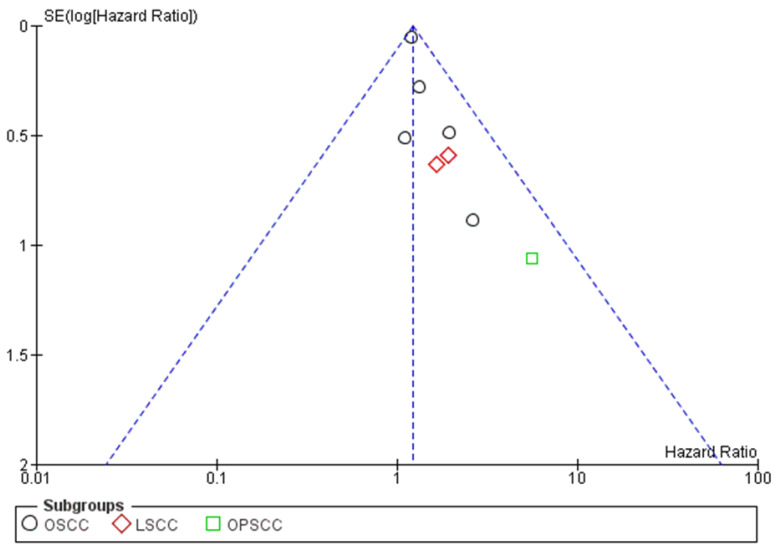
Funnel plot for the meta-analysis. The absence of heterogeneity is graphically highlighted. I^2^ = 0%. SE: standard error. Black circle: OSCC; red diamond: LSCC; green square: OPSCC.

A publication bias assessment was also performed through visual analysis of the distribution of studies on funnel plots, where asymmetry in data distribution was observed (Figure 5).

Furthermore, no elements of heterogeneity were identified, as indicated by the overlapping confidence intervals (Figure 3) and the distribution on the funnel plot (Figure 5).

### 3.6. Trial Sequential Analisis

Trial Sequential Analysis (TSA) was conducted to evaluate the robustness of the meta-analysis results, adjusting them to minimize type I and type II errors. The analysis was performed using Stata 13 ver. 13.1 (StataCorp, College Station, TX, USA) integrated with R 4.2 software (ver. 4.2.1, https://www.r-project.org/, accessed on 10 January 2025) through the “metacumbounds” commands, as described by Miladinovic et al. [44]. The O’Brien–Fleming spending function was applied under a random-effects model.

**Figure 6:**
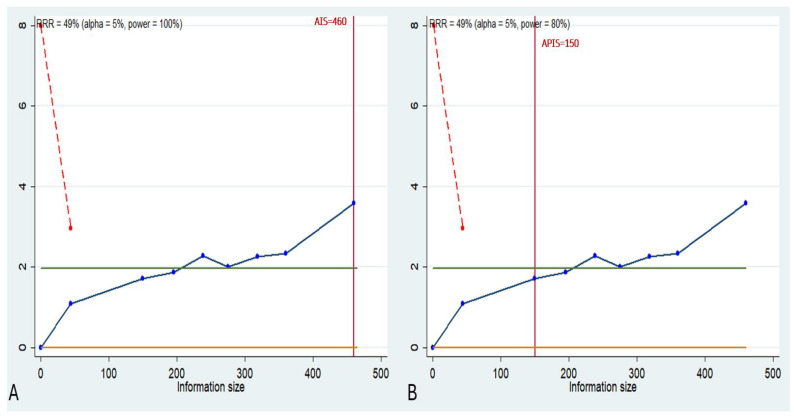
(**A**) AIS, light green line (Z = 1.98); dashed red line (monitoring boundary); blue line (cumulative z curve); red line (sample size). (**B**) APIS, light green line (Z = 1.98); dashed red line (monitoring boundary); blue line (cumulative z curve); red line (sample size).

The APIS (a priori information size) and AIS (accrued information size) commands were utilized via the dialog box to determine the optimal sample size and the power of the results. Assumptions included an average survival rate of 49% [45], a relative risk reduction (RRR) of 48.5%, based on previous studies evaluating prognostic factors in HNSCC [46], an alpha value of 5% (type I error), and a beta value of 20% (type II error). (Figure 6).

The TSA indicates that the expected effect was not observed in the data (survival rate of 49%, RRR 48.5% between the low and high tissue expression groups of miR-375). There is insufficient evidence to confirm the anticipated effect.

The total number of participants required to achieve adequate statistical power was reached. However, the predefined effect was not observed. The result obtained is consistent with a true negative, indicating that no effect is present.

### 3.7. Grade 

The authors also used GRADEpro GDT to assess the quality of the evidence. The results suggest that the quality of evidence is low (Table 4).

## 4. Discussion

A systematic review with meta-analysis and TSA was conducted to explore the potential use of altered miR-375 expression as a prognostic biomarker for survival in HNSCC. This systematic review represents the first meta-analysis with TSA focusing on miR-375 in HNSCC, including eight patient cohorts from six studies, in addition to data from the TGCA cohort, which encompasses approximately 522 HNSCC patients.

A total of 460 patients from the six studies were included in the meta-analysis. To minimize publication bias, gray literature was also searched. TSA results indicated that the meta-analysis, assuming an RRR of 49% between the low- and high-expression groups, did not achieve sufficient statistical power to support its effective prognostic value.

Several studies have confirmed that miR-375 is under expressed in tumor tissues and is downregulated in many solid tumors, indicating that its overexpression is generally associated with tumor growth inhibition [47,48,49,50].

### 4.1. Multifunctional Role of miR-375 in Molecular Regulation, Tumor Invasiveness, and Immune Response in HNSCC

miR-375, initially identified as a pancreatic islet-specific miRNA regulating insulin secretion, and findings highlight that miR-375 is significantly downregulated in various cancers and acts as a suppressor of key cancer hallmarks by targeting critical oncogene [51].

Specifically, miR-375 has been identified as a key regulator of the miR-375/YAP1 molecular axis, influenced by PVT1, an lncRNA that increases YAP1 expression while reducing miR-375 expression [52]. In LSCC, miR-375 targets CST1, inhibiting the aggressive phenotype of tumor cells and promoting apoptosis. It has been hypothesized that the miR-375/CST1 axis may be involved in extracellular matrix degradation, although further evidence is needed [30].

In OSCC, UASR1 acts as a molecular sponge for miR-375, attenuating its inhibitory effects on JAK2 and promoting cell proliferation [53]. Similarly, SNHG17 has been identified as a sponge for miR-375, indirectly promoting PAX6 expression and contributing to tumor proliferation and metastasis [54].

Additionally, miR-375 modulates PD-1/PD-L1 signaling via the JAK2/STAT1 pathway, suggesting its potential as an immune response modulator in HNSCC. This function could have significant implications for targeted therapies aimed at improving immune response efficacy [55]. In addition, miR-375 has also been shown to reduce tumor invasiveness by regulating proteins such as vimentin and L-plastin, which are crucial for cell migration [56]. Moreover, in LSCC, miR-375 and miR-205 act synergistically to regulate invasion and migration via the AKT-mediated EMT pathway, offering new prospects for targeted treatments [57,58].

### 4.2. Expression, Clinical Impact, and Therapeutic Potential of miR-375 in HNSCC

The miR-375 expression is significantly downregulated in tumor tissues compared to normal tissues [59]. This reduction is associated with poor prognosis, metastasis, and increased invasive properties of squamous cell carcinomas [59]. Furthermore, miR-375 suppresses invadopodia activity and extracellular matrix degradation through the regulation of the oncogene AEG-1 [59].

Recent studies have highlighted that miR-375 expression is influenced by nutritional factors. Specifically, it increases with selenium, vitamin C, and vitamin D intake while decreasing with high consumption of added sugars, phosphorus, and vitamin B12. These findings suggest that nutrients may play a crucial role in regulating miR-375 expression, with potential implications for carcinogenesis [60].

Additionally, miR-375 is a promising therapeutic target in HNSCC. Restoring its expression or inhibiting antagonist molecules such as CST1 and SLC7A11 could be an effective strategy for suppressing tumor progression [30,61]. Furthermore, miR-375 has been reported to directly target metadherin (MTDH) in HNSCC, where its downregulation contributes to the oncogenic activity of MTDH [62]. Notably, a study demonstrated that the overexpression of miR-375 in HNSCC cell lines, alongside MTDH knockdown, significantly reduced tumor formation in mice [63], underscoring the critical role of miR-375 in suppressing tumorigenic processes and its potential as a therapeutic target.

Wang et al. [64] demonstrated that miR-375 plays a crucial role in enhancing TNF-α-induced apoptosis in HNSCC cells, showing a significant correlation between its expression and apoptotic sensitivity and demonstrating its applicability in improving chemotherapy efficacy.

The role of miR-375 as a diagnostic and prognostic biomarker, combined with its immune-regulatory implications, emphasizes the need for further studies to fully explore its clinical potential [55].

### 4.3. Meta-Analysis and TSA

The meta-analysis, based on HR for OS, yielded an aggregated HR value of 1.23, 95% CI: [1.10, 1.37], indicating worse survival for patients with low miR-375 expression. The data extracted from Kaplan–Meier curves showed homogeneity with a good overlap of confidence intervals, demonstrating moderate agreement among studies. This was further supported by Higgins’ inconsistency index, which was 0%.

A sensitivity analysis excluding the Yoon et al. study, which had an excessive weight of approximately 92% due to its narrow confidence intervals, resulted in an HR for OS of 1.56, 95% CI: [1.08, 2.26]. Subgroup analysis showed an HR of 1.21 [1.09, 1.36] for OSCC and 1.78 [0.77, 4.15] for LSCC, with low heterogeneity among the three groups (I^2^ = 29.5%).

Data from the TGCA cohort aligned with the meta-analysis findings, showing an HR for OS of 1.32, 95% CI: [0.96–1.8] (Figure 2). These data are statistically significant and support a favorable prognosis for high miR-375 expression.

However, the TSA confirmed that while the number of patients was adequate, the data lacked sufficient statistical power to establish an RRR of 49%. The quality of evidence, as assessed by the GRADE framework, was considered low, primarily due to the retrospective nature of the included studies and the absence of randomized controlled trials (RCTs).

### 4.4. Risk Factors

Examining risk factors (Table 1), miR-375 was found to be overexpressed in all oral samples positive and negative for HPV. According to Harris et al. [33], the frequency of HPV-positive and HPV-negative patients is similar among those with both high and low miR-375 levels.

It is known to be deregulated in pro-inflammatory tissue environments and various cancer types. Notably, miR-375 overexpression inhibits proliferation, migration, and invasion in OSCC. One study confirmed that miR-375 is highly expressed in MCPyV-positive biopsies compared to virus-negative samples [65].

Nicotine may influence miR-375 expression levels. A study by Shen et al. on TCGA-HNSCC and FDEENT-HNSCC revealed reduced levels of miR-375–3p in HNSCC tumor tissues of smokers [50].

### 4.5. Limitations

The limitations of this systematic review are primarily related to the small number of included studies. Additionally, HR values were extracted from Kaplan–Meier curves in three studies using the Tierney method [39], which provides only an estimate of HR and is not exempt from inaccuracies [66,67,68].

The included studies belong to type 2 studies, which primarily examine associations between specific factors and clinical outcomes, as outlined in the PROGRESS guidelines for prognostic research. However, an inherent limitation of these studies is the grouping of patients into “high” and “low” risk categories, without considering individual probabilities of developing clinical outcomes.

In this context, systematic reviews with meta-analyses based on type 2 studies represent only a preliminary step compared to type 3 studies focused on prognostic model research. Currently, no phase 3 studies have explored the relationship between miR-375 and HNSCC.

## 5. Conclusions

This systematic review with meta-analysis and TSA represents the first comprehensive investigation into the potential of miR-375 as a prognostic biomarker for survival in HNSCC. The results suggest that low miR-375 expression is associated with poorer overall survival, as indicated by an aggregated HR of 1.23, though statistical power was limited.

In particular, miR-375 exhibits a multifunctional role in tumor suppression, regulating key molecular pathways, inhibiting tumor invasiveness, and potentially modulating immune responses. Despite its promise, the findings underscore significant limitations, including the small number of included studies, reliance on retrospective data, and heterogeneity in the reported results.

Further research is needed to validate these findings, ideally through larger, well-designed prospective or randomized studies. miR-375 remains a compelling target for therapeutic interventions and a potential prognostic biomarker, warranting continued exploration in the context of HNSCC and other malignancies.

## Figures and Tables

**Table 1:** Data extracted from the 6 studies included, providing information regarding the type of tumor, the location of the tumor, the number of patients with data concerning the average age, the average or maximum follow-up, gender, and the common risk factors in the patients are reported to be smoking, alcohol, and HPV status; Abbreviation: TNM (T: tumor size; N: regional lymph nodes; M: distant metastasis); M (male); F (female); PS (prospective study); RT (retrospective study); TSCC (tongue squamous cell carcinoma). “\” Data not reported in a clear and explicit manner; “+” positive; “−” negative.

First Author, Date	Country	Study Design	Tumor Type and Site	miR	Follow-Up (Months)	Patient		Age (Years)	Smoking			Alcohol			HPV		TNM Stage
						M	F		Yes	No	Ex	Yes	No	Ex	(+)	(−)	
**Zhang et al., 2017 [34]**	China	RT	44 OSCC	miR-375	60	\		\	\	\	\	\	\	\	\	\	\
**Jia et al., 2015 [35]**	China	RT	105 TSCC	miR-375	50	49	56	65 (<60) 40 (≥60)	\	\	\	\	\	\	\	\	59 (I + II), 46 (III + IV)
**Hu et al., 2014 [36]**	China	RT	46 LSCC ^1^	miR-375, miR-21	60	42	4	22 (<65) 24 (≤65)	31	12	\	19	22	\	\	\	31 (I + II), 15 (III + IV)
**Harris et al., 2012 [33]**	USA	PS	123 HNSCC ^2^	miR-375	60	85	38	45 (<58) 38 (59/66) 40 (≥67)	48	18	57	34	89	\	31	74	24 (I + II), 99 (III + VI)
**Hudcova et al., 2016 [37]**	Czech Republic	RT	42 OSCC ^3^	miR-375, miR-29c, miR-200b	48	42	\	63 (47–87)	\	\	\	\	\	\	\	\	18 (T1 + T2), 22 (T3 + T4)
**Yoon et al., 2014 ^4^ [38]**	USA	RT	100 OSCC	miR-375, miR-214-3p	60	61	39	64 (30–83) *	31	61	8	13,	82	6	3	17	58 (I), 42 (II)

^1^ 33 Glottic, 11 Supraglottic, 2 Subglottic; ^2^ (OSCC 43, OPSCC 37, LSCC 43); ^3^ 34 patients included in the analysis; ^4^ In the study by Yoon et al., 2014 [38], as reported in Table 1, there is one additional patient listed under alcohol consumption, resulting in a total of 101 patients compared to the reported total of 100. Additionally, for HPV it is also reported that for 80 patients the HPV status is unknown. However, the data have been presented as they appear in the original study by Yoon et al. * It is a mean calculation, as the study presents the data in the form of an average.

**Table 2:** The values of HR (95% confidence interval) for the different prognostic indices of survival are shown in the table [low expression vs. high expression]. Abbreviations: Overall survival (OS); disease-free survival (DFS); recurrence-free survival (RFS): not available (NA).

First Author, Date	miR	Number (Tumor Site and Type)	OS	DFS	RFS
**Zhang et al., 2017 ^2^ [34]**	miR-375	44 (OSCC)	2.63 (0.46–15.9)	NA	NA
**Jia et al., 2015 ^2^ [35]**	miR-375	105 (TSCC)	1.94 (0.74–5.09)	NA	NA
**Hu et al., 2014 ^2^ [40]**	miR-375	46 (LSCC)	1.66 (0.48–5.72)	NA	NA
**Harris et al., 2012 ^1^ [33]**	miR-375	43 (OSCC)	1.1 (0.4–3.0)	NA	NA
		37 (OPSCC)	5.6 (0.7–44.2)	NA	NA
		43 (LSCC)	1.9 (0.6–6.1)	NA	NA
**Hudcova et al., 2016 [37]**	miR-375	34 (OSCC)	1.32 (0.76–2.27)	1.45 (0.74–2.81)	1.77 (0.67–468)
**Yoon et al., 2014 [38]**	miR-375	100 (OSCC)	1.20 (1.07–1.33)	NA	NA

^1^ The data referring to Harris et al. and reported as OS (overall survival) in this review are labelled as “Overall death” in the original study by Harris et al. However, in the context of that study, they can clearly be interpreted as OS [33]. ^2^ The HR data for OS between low and high expression groups were extracted from Kaplan–Meier survival curves using the Tierney method, as described in Zhang et al., 2017 [34], Jia et al., 2015 [35], and Hu et al., 2014 [40].

**Table 3:** Risk of bias assessment within the studies, with scores ranging from 8 to 10 indicating low quality, 11 to 14 indicating intermediate quality, and 15 to 18 indicating high quality.

First Author, Date	Sample	Clinical Data	Marker Quantification	Prognostication	Statistics	Classical Prognostic Factors	Score
Zhang et al., 2017 [34]	1	1	2	2	2	3	11
Jia et al., 2015 [35]	3	2	2	2	2	3	14
Hu et al., 2014 [40]	1	3	3	2	3	3	15
Harris et al., 2012 [33]	3	3	2	3	3	2	16
Hudcova et al., 2016 [37]	2	2	3	3	3	2	15
Yoon et al., 2014 [38]	3	3	3	3	2	2	16

**Table 4:** Evaluation of GRADEpro GDT; CI, confidence interval; HR, hazard ratio. ⨁⨁ Low.

Certainty Assessment							№ Patients	Effect		Certainty
№ Studies	Study Design	Risk of Bias	Inconsistency	Indirectness	Imprecision	Other Considerations		Relative (95% CI)	Absolute (95% CI)	
**8**	non-randomized studies	not serious	not serious	not serious	not serious	none	452 participants	**HR 1.23** (1.10 to 1.37)	**1 fewer per 1.000** (from 1 fewer to 1 fewer)	⨁⨁

## Data Availability

No new data.

## Abbreviations

HR	Hazard Ratio
OS	Overall Survival
DFS	Disease-Free Survival
RFS	Recurrence-Free Survival
CSS	Cancer-Specific Survival
PFS	Progression-Free Survival
TNM	Tumor, Node, Metastasis (Cancer Staging System)
HNSCC	Head and Neck Squamous Cell Carcinoma
OSCC	Oral Squamous Cell Carcinoma
TSCC	Tongue Squamous Cell Carcinoma
LSCC	Laryngeal Squamous Cell Carcinoma
OPSCC	Oropharyngeal Squamous Cell Carcinoma
HSCC	Hypopharyngeal Squamous Cell Carcinoma
NPC	Nasopharyngeal Carcinoma
miR	MicroRNA
TGCA	The Cancer Genome Atlas
TSA	Trial Sequential Analysis
PRISMA	Preferred Reporting Items for Systematic Reviews and Meta-Analyses
PICO	Population, Intervention, Comparison, Outcome
REM	Random-Effects Model
RCT	Randomized Controlled Trial
SE	Standard Error
CI	Confidence Interval
I^2^	Higgins’ I^2^ statistic (measure of heterogeneity in meta-analysis)
FDR	False Discovery Rate
APIS	A Priori Information Size
AIS	Accrued Information Size
CST1	Cystatin SN
MTDH	Metadherin
JAK2	Janus Kinase 2
STAT1	Signal Transducer and Activator of Transcription 1
PD-1	Programmed Death-1
PD-L1	Programmed Death-Ligand 1
AKT	Protein Kinase B
SNHG17	Small Nucleolar RNA Host Gene 17
UASR1	Upstream Activating Sequence RNA 1
PAX6	Paired Box 6
JAK2/STAT1	Janus Kinase 2/Signal Transducer and Activator of Transcription 1 Pathway
AEG-1	Astrocyte Elevated Gene-1
MCPyV	Merkel Cell Polyomavirus
SLC7A11	Solute Carrier Family 7 Member 11
GRADE	Grading of Recommendations Assessment, Development, and Evaluation
